# Research Involvement, Career Determinants, Study Experiences, and Personality Traits of Health‐Care Students and Workers: The Research In‐Depth Study Protocol

**DOI:** 10.1002/hsr2.72953

**Published:** 2026-08-04

**Authors:** Yassar Alamri, Silvia Caldaroni, Nicole Stormon, Robert Cloninger C, Diann Eley, Erik Monasterio, Danilo Garcia

**Affiliations:** ^1^ Department of General Medicine Christchurch Hospital Christchurch New Zealand; ^2^ Department of Medicine University of Otago Christchurch New Zealand; ^3^ Department of Psychology Sapienza University of Rome Rome Italy; ^4^ Promotion of Health and Innovation for Well‐Being (PHI‐WELL), Department of Psychology University of Stavanger Stavanger Norway; ^5^ School of Dentistry University of Queensland Brisbane Australia; ^6^ Department of Psychiatry Washington University School of Medicine St. Louis Missouri USA; ^7^ Medical School University of Queensland Brisbane Australia; ^8^ Forensic Psychiatrist Christchurch New Zealand; ^9^ Department of Psychology University of Stavanger Stavanger Norway; ^10^ Lab for Biopsychosocial Personality Research (BPS‐PR) International Network for Well‐Being Stavanger Norway; ^11^ Center for Ethics, Law and Mental Health (CELAM) University of Gothenburg Gothenburg Sweden; ^12^ Department of Psychology University of Gothenburg Gothenburg Sweden

**Keywords:** burnout, professional, internship and residency, medical students, personality, vocational guidance

## Abstract

**Background and Aims:**

Medical students and junior doctors undergo rigorous acad emic, clinical and personal demands during early adulthood, a period characterized by identity formation and major life transitions. These pressures contribute to high rates of burnout, diminished motivation and impaired professional development. Personality traits and integrated personality profiles are increasingly recognized as influential factors shaping individuals' responses to stress, sense of vocational calling and career trajectories. However, few studies have simultaneously applied both variable‐centered and person‐centered approaches within a theoretically grounded framework. This study aims to examine how Cloninger's temperament and character dimensions, as well as validated personality profiles, relate to burnout, sense of calling, research proclivity, and career preferences among medical students and junior doctors in New Zealand.

**Methods:**

This cross‐sectional study surveyed medical students from all three campuses of the University of Otago and junior doctors working at Health New Zealand—Christchurch hospitals. Participants completed validated measures of burnout, personality (TCI‐R) and sense of calling, alongside demographic and career preference items. Two‐Step Cluster Analyzes were used to derive temperament, character and integrated personality profiles, constrained to replicate Cloninger's empirically established configurations. Personality profiles were validated using multivariate analyzes of variance. Associations between personality, calling and burnout were examined using correlations, ANOVAs and multiple regressions. Hierarchical regressions assessed whether sense of calling explained additional variance in burnout beyond personality traits.

**Conclusion:**

This study provides a comprehensive investigation of the interplay between personality, sense of calling, and burnout within a large cohort of New Zealand medical trainees. By integrating established personality profiles with contemporary educational outcomes, findings are expected to contribute to a better understanding of the associations between these factors, and to inform future research and educational strategies aimed at supporting well‐being, professional identity and sustainable career development.

## 1 Introduction

Medical training, although rewarding, is among the most physically, emotionally, and psychologically demanding career paths [1]. Medical students and junior doctors face a challenging curriculum alongside long working hours, frequent examinations, the need to stay current with medical literature, and engagement in extracurricular activities to remain competitive [2]. These demands occur during early adulthood, a period of key personal development and identity formation, and are compounded by broader healthcare system pressures, including workforce shortages, limited funding, and increasing clinical complexity driven by technological advances and an ageing population [3, 4]. Given these stressors, it is no surprise that rates of burnout among medical students and doctors are soaring and unlikely to abate without specific interventions [5, 6, 7]. Although reported prevalence estimates vary widely, from 5.6% to 88% depending on geographical context, academic stage, and assessment tool, burnout is consistently linked with negative outcomes, including loss of intrinsic motivation, academic disengagement, thoughts of quitting medical school or work, and reduced social support [7].

A strong sense of vocational calling to medicine has been identified as a protective factor against burnout. Physicians who perceive medicine as a calling report lower odds of burnout and career regret compared with those with a weaker sense of calling [8, 9]. Conversely, disconnection from this sense of meaning, particularly in bureaucratic or dehumanizing clinical environments, has been associated with increased burnout risk [10]. While systemic and institutional stressors significantly contribute to medical student and junior doctor burnout, fatigue, and loss of productivity, personality plays a critical role in shaping how they perceive, interpret and respond to these challenges [11, 12, 13, 14, 15, 16]. Personality traits function as key psychological resources that can either buffer against or amplify emotional strain during medical training.

In medical education research, person‐centered approaches have revealed meaningful sub‐groups of students based upon distinct personality configurations. For example, students with certain personality profiles consistently reported higher well‐being, greater emotional stability, and stronger alignment with people‐oriented career paths [14]. This highlights how meaningful combinations of temperament and character traits can yield richer insights into students' adaptation, motivation, and professional development than analyzes focused on single traits alone. Despite these insights, few studies have integrated variable‐centered (modeling how traits relate to outcomes across variables) and person‐centered (modeling how traits relate to outcomes across individuals) approaches to examine how personality relates to well‐being and vocational motivation in medical education [17, 18]. Moreover, most person‐centered studies rely on exploratory clustering methods rather than theoretically grounded, empirically validated profiles from Cloninger's research, limiting comparability and cumulative value [19, 20, 21, 22, 23].

The Research IN‐DEPTH study addresses this gap by applying a biopsychosocial, person‐centered framework to examine how personality dimensions and profiles relate to burnout and sense of calling among medical students and junior doctors in New Zealand. Grounded in Cloninger's biopsychosocial model of personality [24], it integrates variable‐ and profile‐based analyzes to capture both linear and profile‐specific associations. Conducted in a context of high student distress and a strong focus on socially accountable medical education [25], the study aims to generate robust insights into psychological resilience and vulnerability. Findings may inform strategies to identify at‐risk students, reduce burnout, and support professional development and sense of calling.

## 2 Research Questions and Objectives

### 2.1 Research Questions

1. What is the distribution of personality profiles among medical students and junior doctors?
2. How, if at all, do medical students and junior doctors with certain personality profiles differ in their levels of burnout and sense of calling?
3. How are personality profiles associated with students' and junior doctors' inclination towards certain specialties or research activities?
4. Which personality profiles identify medical students and junior doctors at greatest risk of burnout and reduced productivity?

### 2.2 Study Objectives

1. Examine linear relationships among personality dimensions, sense of calling and burnout in New Zealand medical students and doctors.
2. Identify the prevalence of empirically validated personality profiles among New Zealand medical students and doctors using the Temperament and Character Inventory–Revised (TCI‐R).
3. Test whether sense of calling and burnout differ significantly among students and doctors with distinct personality profiles.
4. Investigate which personality dimensions are associated with sense of calling and burnout within each personality profile.
5. Evaluate whether sense of calling explains additional variance in burnout within each personality profile beyond personality dimensions.
6. Investigate career preferences of New Zealand medical students and junior doctors, and whether demographic and personality factors are associated with these.
7. Assess whether research proclivity and involvement differ significantly among students and doctors with distinct personality profiles.

## 3 Methods

### 3.1 Definitions

Burnout is conceptualized as a syndrome or state of emotional and physical exhaustion resulting from workplace stress [26, 27]. The sense of vocational calling is often defined as a deep‐seated belief that one's work is meaningful, socially valuable, and personally fulfilling [28]. Burnout among medical students and trainees has been linked to serious negative consequences; for example, higher burnout levels independently predict thoughts of dropping out of medical school, and students with burnout tend to exhibit reduced empathy and professional disengagement [7]. By contrast, a strong sense of calling to medicine tends to be protective and motivating—students who report a calling show higher well‑being, life satisfaction and motivation, and they are more resilient in their studies and patient care. Indeed, calling correlates positively with empathy and can buffer the harmful effects of burnout [29, 30]. Cloninger's biopsychosocial model of personality (see below) helps explain these findings. In this framework, traits such as high harm avoidance (a temperament trait) and low self‑directedness and cooperativeness (character traits) predict higher academic burnout [31], whereas the opposite profile—low harm avoidance together with high self‑directedness (and self‑transcendence)—is associated with greater resilience and a strong vocational calling [32].

According to Cloninger [24], human personality reflects the interplay of three major systems of learning and memory that have developed over a long sequence of evolutionary steps. The first is the procedural system, responsible for automatic emotional responses, such as anger, fear, disgust, and ambition, shaping our likes and dislikes or temperament. The second, the propositional system, supports intentional self‐direction and cooperation within social environments, enabling individuals to align behavior with their own goals and purpose, and shared values and norms. The third system, uniquely present in humans [22], is the episodic system, which underlies our capacity for self‐awareness, introspection, and recollection of autobiographical memories. Together, the propositional and episodic memory systems comprise our character, which is what we make of ourselves intentionally. Put another way, our character allows us to consciously regulate the habits and emotional reactivity of our temperament to behave in accordance with our values and goals [33, 34, 35].

Hence, according to Cloninger's biopsychosocial model, personality consists of four temperament dimensions and three character traits. The four temperament dimensions reflect moderately heritable and stable emotional response patterns: (1) Novelty Seeking, the tendency toward frequent activation or initiation of behaviors in response to novel stimuli, even if they are not predictive of reward, expressed as frequent exploration of new unfamiliar places or situations, quick loss of temper, impulsive decision making, and active avoidance of monotony; (2) Harm Avoidance, the tendency to avoid or cease behaviors in response to signals of punishment or loss of reward (i.e., aversive stimuli), expressed as fear of uncertainty, shyness of strangers, quick fatigability, and pessimistic worry about future problems; (3) Reward Dependence, the sensitivity to signals of reward, particularly social approval, expressed as sentimentality, social attachment, and dependence on the approval of others; and (4) Persistence, the tendency to persevere despite fatigue or frustration, overachieving, and perfectionism [19]. The three character traits, though as heritable as temperament, develop through life experience via epigenetic mechanisms and reflect individual differences in goals, values, and self‐concept: (1) Self‐Directedness, the ability to control, regulate, and adapt behavior in accordance with their own goals and values, involving traits such as responsibility, purposefulness, and self‐acceptance; (2) Cooperativeness, the extent to which a person identifies with and accepts others, expressed through tolerance, empathy, and helpfulness; and (3) Self‐Transcendence, our capacity for spirituality, selflessness, patience, and identification with something greater than the self that gives meaning to one's existence, such as humanity, nature, God, or the universe [36, 37].

Regarding temperament, three heritable profiles have been identified [19]. The “Reliable” temperament profile is characterized by low in Novelty Seeking (i.e., deliberate, thrifty, and orderly), low in Harm Avoidance (i.e., optimistic, confident, outgoing, and vigorous), high in Reward Dependence (i.e., sentimental, friendly, and approval seeking), and high in Persistence (i.e., determined). The “Sensitive” profile is characterized by high in Novelty Seeking (i.e., impulsive and extravagant), high in Harm Avoidance (i.e., pessimistic, fearful, shy, and fatigable), and high in Reward Dependence (i.e., sentimental and friendly). The “Antisocial” profile is characterized by high in Novelty Seeking (i.e., extravagant, rule‐breaking but not inquisitive), low in Reward Dependence (i.e., cold, detached, and independent), and low in Persistence (i.e., easily discouraged).

Regarding character, five heritable profiles have been identified [20]. The “Resourceful” character profile is characterized by high in Self‐Directedness only and includes individuals who are purposeful, responsible, and able to regulate their behavior in accordance with their own goals. While these individuals tend to be independent and resilient, their lower levels of social connectedness and spiritual openness may limit broader sources of meaning and prosocial engagement. The “Organized” profile, characterized by high Self‐Directedness and Cooperativeness, but low Self‐Transcendence, included individuals who are both self‐reliant and socially responsible. These individuals are often dependable and community‐oriented but may struggle to connect with broader existential or spiritual aspects of life, potentially limiting their capacity for deeper meaning‐making. The “Creative” profile, characterized by high levels in all three character dimensions, presents the most psychologically mature and integrated character configuration. These individuals are typically autonomous, compassionate, and capable of experiencing a strong sense of unity with something beyond the self. The “Dependent” profile, characterized by high Cooperativeness but low Self‐Directedness and Self‐Transcendence, included individuals who are warm and agreeable but often lack self‐acceptance, purpose, and meaning. They may rely heavily on others for direction and validation, which can lead to vulnerability under stress or in environments lacking strong external guidance. Lastly, the “Apathetic” profile, characterized by low levels in all three character traits, included individuals who experience low self‐esteem, social detachment, and existential emptiness, reflecting a lack of self‐agency, empathy, and meaning.

Building on this theoretical framework, Cloninger and colleagues recently validated distinct personality configurations, each associated with largely non‐overlapping sets of genes, in culturally diverse samples. They identified three nearly distinct joint personality networks in their pooled community sample across Finland, Germany, and South Korea (*N* = 2,078), by combining the previously validated temperament and character profiles**: the “Creative/Reliable” profile (38.55%), the “Organized/Reliable” profile (32.43%), and the “Emotional/Unreliable” profile (29.02%) [21]. These joint profiles demonstrated complex internal interactions between temperament and character dimensions. Importantly, the “Creative/Reliable” profile was associated with the highest levels of well‐being, but also a slightly elevated risk of ill‐being compared with the “Organized/Reliable” profile. In contrast, individuals within the “Emotional/Unreliable” profile exhibited the highest level of ill‐being and the lowest level of well‐being. Remarkably, these three phenotypic personality profiles mapped onto three corresponding environmental networks, each associated with unique constellations of life experiences and single‐nucleotide polymorphism sets (SNPs). This one‐to‐one correspondence provides strong support for a dynamic biopsychosocial model, in which well‐being, dysfunction, and suffering emerge not solely from trait constellations but from the interaction of genetic predispositions with environmental contingencies. From this perspective, as originally proposed by Cloninger, personality development reflects an evolving adaptive system in which certain configurations may predispose individuals either to resilience and thriving under personally relevant supportive conditions, or to psychological vulnerability under chronic or adverse stress [21].

### 3.2 Study Setting and Design

This is a cross‐sectional study, based in New Zealand. It sets out to capture the experiences of all current medical students at the University of Otago's campuses (Christchurch, Dunedin, and Wellington), as well as the experiences of junior doctors working at Health New Zealand—Christchurch hospitals (Ashburton Hospital, Burwood Hospital, Christchurch Public Hospital, and Hillmorton Hospital).

### 3.3 Inclusion and Exclusion Criteria

Eligible students were all current medical students at the University of Otago (in all three campuses) at the time of the survey. Additionally, all junior doctors working at the various hospitals affiliated with Health New Zealand—Christchurch were eligible to participate. Participants outside these institutions and those younger than 18 years were not eligible to participate.

### 3.4 Recruitment

Participants were invited to complete an online questionnaire via email over a 16‐week period. Three reminder emails requesting participation were sent at 4‐week intervals. In addition, snowball sampling was utilized in order to increase the response rate.

### 3.5 Study Instruments

The questionnaire included demographic questions (age, sex, ethnicity and marital status), academic details (current position, prior academic degrees, and envisaged career plans), and participants' top three specialty preferences. The Maslach Burnout Inventory [26, 27] was used to measure participants' experiences of occupational burnout. The shortened 15‐item inventory used a five‐point Likert scale (1 = not at all to 5 = very often). A total score was calculated by summing the 15 items.

Temperament and character dimensions were measured using the 140‐item TCI‐R developed by C. Robert Cloninger [36, 37]. Items were rated on a 5‐point scale from 1 (“Definitely False”) to 5 (“Definitely True”). The four temperament dimensions are: Novelty Seeking (e.g., “I often try new things just for fun or thrills, even if most people think it is a waste of time”), Harm Avoidance (e.g., “I often feel tense and worried in unfamiliar situations, even when others feel there is little to worry about”), Reward Dependence (e.g., “I like to discuss my experiences and feelings openly with friends instead of keeping them to myself”), Persistence (e.g., “I often push myself to the point of exhaustion or try to do more than I really can”). The three character dimensions are: Self‐Directedness (e.g., “In most situations my natural responses are based on good habits that I have developed”), Cooperativeness (e.g., “I often consider other persons' feelings as much as my own”), and Self‐Transcendence (e.g., “I sometimes feel so connected to nature that everything seems to be part of one living organism”). For each dimension, item scores were summed and divided by the number of items in that dimension, yielding mean scores. Higher mean scores indicate stronger expression of the corresponding trait. Internal consistency (Cronbach's alpha) in the present study was 0.75 for Novelty Seeking, 0.93 for Harm Avoidance, 0.83 for Reward Dependence, 0.89 for Persistence, 0.88 for Self‐Directedness, 0.87 for Cooperativeness, and 0.89 for Self‐Transcendence.

Sense of calling was measured by the shortened two‐item version drawn from the Brief Calling Scale [28]. The shortened version demonstrated similar reliability to the full Brief Calling Scale whilst reducing the number of questionnaire items to be answered by the participants [28]. Students rated agreement with the two items (“I feel I have a calling to be a doctor” and “I have a good understanding of my calling as a doctor”) on a 5‐point scale (1 = “Not at all true of me” to 5 = “Totally true of me”). The two items were summed and divided by two to yield a mean score, with higher scores indicating a stronger sense of calling (Cronbach's alpha = 0.78).

### 3.6 Data Collection

The study questionnaire was administered through the SurveyMonkey platform for electronic delivery. Eligible participants were invited to participate via an email from institutional representatives (University of Otago and the Resident Doctors' Support Team at Christchurch Hospital) including a brief synopsis of the study. Live links to the participant information sheet and to the survey were provided. As the survey was confidential, it was impossible to completely avoid the risk of multiple responses. However, participants were instructed to complete it only once, and not to share the link with others.

### 3.7 Data Analysis

Statistical analyzes were conducted using IBM SPSS Statistics (Version 30). Responses with > 5% missing values were excluded from analyzes. The pattern of missing data was examined using Little's Missing Completely at Random (MCAR) test; given the low proportion of missingness, listwise deletion was applied. Before inferential analyzes, statistical assumptions were assessed. Univariate normality was evaluated through skewness and kurtosis values and Shapiro–Wilk tests; homogeneity of variance was assessed with Levene's test, and homogeneity of covariance matrices for the MANOVAs with Box's M test. For all regression models, multicollinearity was evaluated using variance inflation factors (VIF < 5) and tolerance statistics, and model diagnostics were inspected, including normality and homoscedasticity of residuals (via residual‐vs.‐fitted plots) and influential cases (via Cook's distance). Pearson's correlation coefficients were computed to examine the linear relationships among participants' scores on temperament and character dimensions, burnout, and sense of calling.

Next, we identified latent personality configurations using a series of Two‐Step Cluster Analyzes based on participants' scores on the seven personality dimensions. The clustering procedure followed a three‐stage process, consistent with prior molecular and phenotypic clustering research by Cloninger and colleagues. More specifically, the number of clusters extracted at each stage was theoretically constrained to replicate the biologically and phenotypically validated configurations established by Cloninger and colleagues [19, 20, 21, 22, 23, 38, 39]. This theory‐ and evidence‐driven approach was intended to enhance interpretability, facilitate replication, and ensure alignment with the biopsychosocial and dynamic structure of personality. Constraining the number of clusters to replicate Cloninger's validated configurations prioritized theoretical alignment and cross‐study comparability over purely data‐driven exploration; these solutions were therefore treated as confirmatory rather than exploratory. To assess robustness, we conducted unconstrained Two‐Step Cluster Analyzes, with the number of clusters determined automatically by the Bayesian Information Criterion, as a sensitivity analysis, and compared the resulting cluster composition and interpretability with the theoretically constrained solutions.

In the first stage we constrained cluster extraction to three Temperament Clusters based on continuous scores on the four temperament dimensions. In the second stage, we constrained cluster extraction to five Character Clusters based on continuous scores on the three character dimensions. In the final stage, we constrained cluster extraction to three integrated Personality Clusters using the categorical three temperament and five character profile memberships derived from the previous stages. After this, we conducted two one‐way multivariate analyzes of variance (MANOVAs) to validate the resulting Personality Clusters. The first MANOVA tested differences in the four temperament dimensions across the Personality Clusters, and the second tested differences in the three character dimensions. After validating the clusters (i.e., personality profiles), we conducted two one‐way ANOVAs to examine whether burnout and sense of calling differed significantly across the personality profiles. All analyzes of variance included Bonferroni‐corrected post‐hoc tests when appropriate.

Moreover, to examine which personality dimensions were associated with levels of sense of calling and burnout within each of the three personality profiles, we conducted separate multiple regression analyzes using the seven TCI dimensions as predictors and sense of calling as the outcome variable in one set of regressions and burnout in the other. To further explore the additional value of sense of calling in explaining burnout, we conducted additional hierarchical regression analyzes, for each personality profile, with personality dimensions entered in the first step, and sense of calling entered in the second step.

## 4 Ethical and Cultural Considerations

### 4.1 Ethical Considerations

The study protocol was approved by the University of Otago Human Ethics Committee (reference D18/207). Participation was entirely voluntary, and refusal to participate did not affect the person in any way. If a participant agreed to take part, they were still free to withdraw from the study at any time, without having to give a reason. Ten respondents, chosen at random, received a $20 supermarket voucher. Participants did not otherwise personally benefit other than receiving their individual TCI results.

### 4.2 Responsiveness to Māori

Consultation with Māori researchers was undertaken as part of the ethics application. The project followed local guidelines that aim to ensure that research activities contribute to Māori health advancement [40].

### 4.3 Managing Risks to Participants

Mental risk of learning about one's personality is possible, but there are no “good” or “bad” traits in the TCI. Participants were provided with information regarding options for professional support, including the contact details of the ethics committee's administration; issues raised were treated in confidence and investigated, and the participant was informed of the outcome.

Theoretical breach of confidentiality is another risk. However, the research team can only view responses in aggregate form. Demographic information is not linked to participants' responses.

## 5 Dissemination Plan

Results will be disseminated as peer‐reviewed journal publications, as well as conference and symposia presentations. Additionally, findings will be communicated to relevant university and hospital stakeholders, including University Deans and hospital senior leadership.

### 5.1 Study Status

Data collection for the study has been completed, and data analyzes are currently underway. Over the study period, approximately 1473 medical students were enrolled in the MBChB program at the University of Otago [41]. Of those, 588 completed the survey, yielding a response rate of 39.9%. Over the same period, the total number of junior doctors employed by Health New Zealand—Christchurch hospitals was 511 (Karl Haase, Resident Doctors’ Support Team, personal communication), a minority of whom (number undisclosed for confidentiality purposes) would have been on extended leave (e.g., parental, sickness, and leave‐without‐pay). Of those, 127 responded to the survey (giving an estimated response rate of 24.9%).

## 6 Discussion

### 6.1 Contributions

This large cross‐sectional study aims to provide understanding of the interplay between personality and various current and future career aspects pertaining to New Zealand medical students and junior doctors. Clearer understanding of the role of personality may assist in identification and early intervention for clinicians at risk of burnout and to improve satisfaction and effective functioning. The Research IN‐DEPTH team's collective expertise, diversity, and experience will enable the production of a substantial body of novel knowledge that will enhance understanding within the New Zealand context. Additionally, it will contribute valuable insights to the international discourse on personality influences on student and junior doctor career choices and research endeavors.

### 6.2 Limitations

Given the cross‐sectional, observational design and reliance on self‐reported measures, the findings should not be interpreted as evidence of causal relationships or predictive validity. Future longitudinal studies may help clarify the directionality of these associations, and evaluate whether these factors may be useful in identifying students potentially vulnerable to burnout and in informing targeted interventions. Additionally, since participants who chose to complete the survey may have differed systematically from non‐respondents—including in their levels of burnout, wellbeing, or interest in the study topic—and represented only 39.9% of the eligible students and 24.9% of eligible doctors, the findings should be interpreted with caution as they may not be generalizable to the broader population of medical trainees. In addition, constraining the cluster solutions to replicate Cloninger's empirically validated configurations, although it enhances cross‐study comparability and interpretability, may limit the detection of novel, sample‐specific profiles and could favor the confirmation of expected structures; we therefore complemented the constrained solutions with unconstrained sensitivity analyzes (see Data analysis). Finally, the study utilized a shortened measure of calling rather than the full instrument. Although this approach reduces respondent burden and likely improves survey completion rates, abbreviated measures may not fully capture the multi‐dimensional nature of calling and may be associated with lower reliability. Consequently, some aspects of the construct may be under‐represented, and the strength of observed associations may be attenuated. Future studies could employ more comprehensive measures to further explore specific dimensions of calling and their relationships with personality and burnout.

## 7 Conclusions

We describe the protocol of a large cross‐sectional study that investigates the interplay between personality and various current and future career aspects pertaining to New Zealand medical students and junior doctors. Ethical approval has been obtained following Māori consultation in order to ensure cultural responsiveness. Data collection has been completed, and analyzes are currently under way. Accurate and rigorous reporting of the study's findings will be ensured by following international guidelines, including the STROBE framework [42]. It is hoped that our findings will provide the evidence‐base for solutions that would aid university and hospital policy‐makers and stakeholders in helping medical students and junior doctors at risk of burnout and loss of productivity.

## Author Contributions

**Yassar Alamri:** conceptualization, writing – original draft, methodology, data curation. **Silvia Caldaroni:** writing – review and editing, formal analysis. **Nicole Stormon:** formal analysis, writing – review and editing. **C. Robert Cloninger:** writing – review and editing, formal analysis. **Diann Eley:** formal analysis, writing – review and editing. **Erik Monasterio:** conceptualization, funding acquisition, supervision, writing – review and editing. **Danilo Garcia:** writing – review and editing, resources, supervision, formal analysis.

## Ethics Statement

This study was approved by the University of Otago Human Ethics Committee (reference D18/207). All participants provided consent on the electronic survey form.

## Consent

Participants consented for anonymised data publication.

## Conflicts of Interest

The authors declare no conflicts of interest.

## 1 Transparency Statement

Yassar Alamri and Danilo Garcia affirm that this manuscript is an honest, accurate, and transparent account of the study being reported; that no important aspects of the study have been omitted; and that any discrepancies from the study as planned (and, if relevant, registered) have been explained.

## Data Availability

The data that support the findings of this study are available from the corresponding author upon reasonable request. The datasets used and/or analyzed during the current study are available from the corresponding author on reasonable request after completion of data publication.
